# EU resilience in times of COVID? Polity maintenance, public support, and solidarity

**DOI:** 10.1057/s41295-023-00327-7

**Published:** 2023-06-12

**Authors:** Ioana-Elena Oana, Stefano Ronchi, Zbigniew Truchlewski

**Affiliations:** 1 European University Institute, Fiesole, Italy; 2 University of Milan, Milan, Italy; 3 European University Institute, Fiesole, Italy

## Abstract

This introduction presents the theoretical framework, aims, and summary of this special issue. We want to explain the European Union’s (EU) response to the COVID crisis from a ‘polity perspective’ (Kriesi 2021; Ferrera 2005). We conceptualize the EU as a compound ‘experimental’ polity which develops along three dimensions: binding (capacity building and sovereignty), bounding (bordering), and bonding (solidarity and loyalty). We structure the contributions around the following themes: *polity building and polity maintenance* (how did COVID affect policymaking in the EU?); *reactions to polity building: public support, populism, and emergency politics* (did the European public perceive emergency politics as illegitimate? did the EU’s policy response spur populism?); and *solidarity and bonding* (to what extent did the crisis stimulate cross-national solidarity?). We show that, overall, the EU weathered the COVID storm better than expected for a potentially fragile multilevel polity. The crisis triggered unprecedented institutional innovation, underpinned by pan-European solidarity, and EU citizens did not backlash against emergency politics.

## Introduction

Since 2008, the EU has faced a series of major crises—Eurozone, refugees, Brexit—which have tested its resilience as a *sui generis* multilevel polity. On the one hand, crises can pose a threat to the EU: member states can fall back on national demarcations, undermining European bonds of solidarity and public support. On the other, crises can stimulate policy responses and spur institutional development, resulting in a more resilient polity. The multi-faceted nature of the COVID-19 pandemic makes it an essential case for investigating tensions between threat and resilience. COVID-19 played out as both a public health and a socio-economic crisis, involving multiple problem pressures, and generating various socio-spatial (a) symmetries across the EU.

Influential accounts of European integration and its developments have mostly focused on the many hurdles to EU policymaking, thus being critical of the EU’s capacity to respond to any crisis (Jones et al. 2021), not least owing to recurring ‘joint decision traps’ in inter-governmental bargaining (Scharpf 2006) and to its high vulnerability to politicization in national electoral arenas (Hooghe and Marks 2009). In this special issue, we take a step back. We seek to explain the responses of the EU and its member states to these COVID crises from a *polity perspective*. Inspired by a research tradition which dates back to the Rokkan’s seminal work on state formation (Rokkan 1974), we focus on the complex structuring of the EU as a compound ‘experimental’ polity (Ferrera et al. 2023; Bartolini 2005; Ferrera 2005). In so doing, we inspect the politics of crisis policymaking and how it impacts the resilience of the EU understood in this way.

This special issue investigates how the EU has fared during COVID on two fronts. We appraise its policy reach and functional effectiveness, but we also consider questions of political cohesion, which in a multilevel polity ultimately rest on cross-national solidarity. Broadly speaking, the special issue asks how the COVID pandemic and ensuing emergency politics affected these fundamental dimensions of the European polity—binding (institutional competences and capacities, political support for supranational coordination), and social bonding (solidarity) between citizens of different member states.

More specifically, the contributions to this special issue address the following questions:

- How did the COVID crisis affect the politics of policymaking in the EU? In what ways did political leaders engage in ‘polity maintenance,’ working to defuse polity threats and build resilience?
- How did the European public react to the executive-led ‘emergency politics’ mode of crisis governance?
- What kinds of conflicts emerged during the pandemic between and within member states and to what extent, if at all, did they undermine public support for the EU and cross-national solidarity?

## The EU polity perspective: bonding, binding, and bounding

The EU polity has emerged as a *sui generis* multilevel polity, which has brought together previously autonomous member states. This feature makes the EU experimental in the sense that its developments can hardly be predicted using well-known types of polity such as federations and nation states as yardsticks (Kriesi et al. 2021). To analyze its nature and developments, following Ferrera (2005), we break down the EU polity into three essential dimensions: The demarcation of territories and their citizens and deciding who has access to the collective public goods (*bounding*), the scope of authoritative control over such units, problem solving capacities and competences within the polity (*binding*), and solidarity building and strengthening of shared identity (*bonding*). The EU’s political–economic system, we suggest, has no given *finalité*, be that in the form of a federal state (Fabbrini 2005) or in a continued ‘rescue of the nation state’ (Milward 2000).

By polity building, we mean significant strengthening of the polity through any one of the three Bs. In contrast to nation states, the EU does not necessarily react to crises by centralizing and monopolizing capacity toward the center. It can, instead, create *joint* capacity, managed by European institutions while member states retain budgetary control and veto power. This special issue shows that such a ‘weak centre’ can have its strengths (Alexander-Shaw, Ganderson and Schelkle, 2023). The combinations of the three Bs imply that crises can spur polity development in multiple possible directions, thereby creating a ‘garden of forking paths’. The choice of the path taken at each fork will depend on many conditions, from the crisis problem pressures, to coalitional dynamics, and leadership, to name but a few. As a result, crises push the EU into institutional ‘layering’ and ‘redirection’ (Streeck and Thelen 2005). All in all, the polity perspective provides an innovative and flexible analytical framework to shed light on how the EU works and how it can react to crises.

## EU polity maintenance and the COVID crisis

The developments of the multilevel EU polity can also lead to fragile institutional equilibria. Moreover, the lack of direct democratic mechanisms to effectively channel dissensus makes the EU particularly vulnerable to politicization (Mair 2007). The preservation of such a complex multilevel polity, therefore, becomes a particularly demanding political endeavor, requiring an extraordinary ‘polity maintenance’ effort in times of crises that escalate into politicization of the polity itself.

By polity maintenance we mean ‘a deliberate strategy driven by the primary objective of safeguarding the polity as such’ (Ferrera et al. 2023: 1331). To the extent that they recognize the benefits from EU integration as a common good, national politicians pursuing polity maintenance subordinate narrower policy aims or electoral strategies to the ultimate objective of preserving the stability of the EU. The need for polity maintenance is particularly high when severe socio-economic shocks can escalate into fully fledged political conflicts that question the viability of the polity itself. COVID is a case in point: The pandemic forced lockdowns and border closures, thus risking to jeopardize the ‘bounding’ foundations of the EU as well as the freedoms on which the Single Market and Schengen are built. The COVID crisis was very much an outlier in terms of magnitude, speed, and distributional implications. It materialized as a sudden and unexpected shock, with a high degree of urgency and uncertainty that brought about exceptional salience. Contrary to previous crises, much of the world came to a stop in a matter of days in March 2020 and policymakers had to scramble to respond (Kriesi and Bojar, 2023). This unprecedented dual crisis structure (slow burning economic and fast burning health crises—Truchlewski et al. 2021) and the dependence on executive and emergency politics for handling it are some of the aspects explored in this special issue.

Moreover, COVID was arguably a symmetric shock that hit all member states, even if the consequences of the shock still varied across them (Ferrara and Kriesi 2021). Such a crisis can cause two types of reactions: ‘every state for itself’ or ‘one for all and all for one.’ The former was the case in the eurozone crisis, whereby member states’ diverging views and economic interests carried the day. The findings of this special issue tell a different story for the COVID crisis. In short, we show how ‘bonding’ went along with ‘binding’: high levels of political support and cross-national solidarity gave policymakers more room for maneuver to pull Europe from the brink of disintegration by advancing new far-reaching policy solutions (above all, NGEU) without compromising the legitimacy of the EU solidarity.

## Lessons from the COVID crisis for the European Polity

The EU polity perspective shows how polity maintenance efforts played out across the three Bs, both on the supply (decision-making) and on the demand-side of politics (public reactions). Therefore, the special issue is structured along the following lines: the first two papers look at the supply-side of EU politics, asking how the unique characteristics of both the COVID crisis and the EU have shaped decision-making (binding and bounding) during the pandemic with reference to polity maintenance and building. The other contributions turn to political demand (bonding and public support for the EU). Three papers examine how the public reacted to the emergency politics triggered by COVID and whether this was an opportunity for populism to thrive. The last two papers look at how much support for EU bonding emerged, focusing on cross-national solidarity and its micro-foundations.

### Binding: polity building and policy maintenance

The first paper, by Kriesi and Bojar 2023, compares the COVID and the refugee crises to shed light on the role played by different crisis situations in shaping decision-making. In doing so, it focuses on the (a) symmetries in the distribution of crisis pressures and of competences in the multilevel EU polity. Empirical analysis reveals similarities between the crises, such as intense polarization and conflict, executive dominance, and the importance of coalitions of critical member states for the success of new policy initiatives. However, it also brings to the fore consequential distinctions. The higher urgency of the problem pressure in the COVID crisis, its more limited politicization of national identities, and the more symmetric distribution of its consequences activates the potential of the weak center and facilitates a joint solution put forward by the core coalition in the crucial fiscal policy domain.

The second paper, by Alexander-Shaw, Ganderson, and Schelkle (2023), compares EU crisis politics with the USA, showing how the unique characteristics of the EU experimental polity incentivize polity maintenance. Despite the EU possessing a ‘weak centre’ for executive governance—as opposed to the USA—the functional performance of both were comparable in the first phase of the pandemic. The EU demonstrated a paradoxical strength: The risk for a policy crisis to escalate into an existential crisis triggered political responses driven by a polity maintenance rationale, which concentrated leaders’ minds on overcoming polarization and forging compromise.

### Reactions to polity building: emergency politics, public support, and populism

By stretching constitutional norms and decision-making procedures, emergency politics may have corrosive effects on democratic processes. In this light, the paper by Ganderson, Schelkle, and Truchlewski (2023) analyzes the European public’s perception of COVID’s acute emergency politics. The results generally reveal low levels of concern among the public and cast doubt on the claim that crisis management might be fostering Euroscepticism. On the contrary, the authors suggest that integration–demarcation attitudes seem to either contain concerns rather than create new divides (in the case of pro-integration respondents) or reinforce (for pro-demarcation respondents) concerns regarding crucial features of EU crisis management.

Complementary to these results, the paper by Wang, Bojar, Oana, and Truchlewski (2023) looks at the ‘real-time effects’ on support for the EU of key emergency decisions taken by European institutions. Using social media data, the results show that these key decisions increase pro-EU sentiment and reduce public polarization. Hence, emergency politics not only results in a rally around the national flag, but also in a rally around the *European* flag.

Finally, Oana and Bojar (2023) paper looks at the implications of crisis management for the success of populist forces in Europe. The results show that even if the salience of the COVID issue marginalized those issues on which the long-term successes of populist parties in Europe have been capitalizing (especially immigration), novel conspiracy beliefs reinforced populist and anti-technocratic attitudes. Nevertheless, crisis management played an important role in the conspiracy-populism nexus: The magnitude of this effect was conditional on the way governments—and, to a lesser extent, EU institutions—were able to handle the crisis.

### Bonding and cross-national solidarity

The paper by Kyriazi, Pellegata, and Ronchi (2023) investigates whether and how the COVID crisis moderated public support for cross-national fiscal solidarity in the EU. Based on survey data from 2019 to 2020, their results indicate that average levels of support for European solidarity during the first wave of COVID did not substantially differ from ‘normal’ times. Nevertheless, the traditional drivers of solidarity—identification and self-interest—lost importance. In a ‘common crisis’ situation, fiscal solidarity can also find some support among those identifying exclusively as nationals and among ‘self-interested’ individuals.

These findings resonate with those by Russo (2023), who shows that the COVID pandemic contributed to the emergence of a distinct supranational solidaristic space. The individual motivations behind supranational solidarity, however, appear utilitarian in nature, rather than based on sentiments of a ‘community of fate’. In a compound, experimental polity as complex as the EU, solidarity leads back to reciprocity, in that citizens’ expectations of net benefit/loss for their country matter a great deal. This has important implications for polity building. As long as the EU is seen as beneficial to its members, polity maintenance efforts may rest on a permissive consensus. Nevertheless, not even a truly common crisis such as COVID had the power to turn the EU into a fully fledged ‘community of fate.’

## Ways forward for the EU-polity

Our polity argument adds a new layer of supply-side EU politics (polity maintenance) on top of existing approaches that mostly deal with demand-side domestic electoral backlashes (e.g., postfunctionalism). Moreover, instead of focusing on single policy responses, we look at crises’ systemic implications for the EU polity as a whole.

Despite the intensity of the COVID crisis, the EU’s ‘weak center’ held strong against the headwinds, and EU citizens, at least in the initial phases of the pandemic, did not backlash against emergency politics. Notwithstanding the initial fall back on re-bordering due to the unprecedented public health challenges, the EU’s binding structure has weathered the storm much better than expected from a fragile multilevel polity. Moreover, crisis policymaking was enabled by robust cross-national bonds of solidarity.

The novel approach taken by this special issue opens avenues for future research in at least two ways. The recent transformations of the EU along a peculiar combination of binding, bounding, and bonding may have eventually broken the path dependence of previous EU crises (Schelkle 2021). Compared to the Eurozone crisis and its austerity politics, the policy responses to COVID seem today much more aimed at leveling the playing field in which member states find themselves, which may explain why emergency politics were not rejected and a modicum of cross-national solidarity was created. At the same time, however, the solutions put forward are temporary (e.g., NGEU, SURE), and they did not bring about fully fledged constitutional changes. They are the result of a contingent and, perhaps, fragile political compromise, while the EU is still fraught with profound economic and social imbalances. The jury is still out on whether the COVID crisis has indeed opened the window to an enduring deepening of European integration. Our special isssue establishes theoretically and empirically grounded evidence, but in a time of extraordinary uncertainty. It remains to be seen whether, in the medium-to-long run, EU citizens will remain satisfied by the new arrangement of the three Bs. On the one hand, the conflicts that erupted around the third wave of lockdowns and vaccinations, as well as around the conditionality around NGEU, cast a shadow of pessimism over these prospects. On the other hand, the latest crisis embroiling the EU since the full-scale Russian invasion of Ukraine might bring about new configurations of the three Bs which future research should address. While an external threat can provide a strong impetus for centripetal binding and solidarity, the energy crisis hitting COVID-scarred European economies might trigger centrifugal forces in the long run.
